# Uptake and safety of pneumococcal vaccination in adults with immune-mediated inflammatory diseases: a UK wide observational study

**DOI:** 10.1093/rheumatology/keae160

**Published:** 2024-03-13

**Authors:** Georgina Nakafero, Matthew J Grainge, Tim Card, Christian D Mallen, Jonathan S Nguyen Van-Tam, Abhishek Abhishek

**Affiliations:** Academic Rheumatology, School of Medicine, University of Nottingham, Nottingham, UK; Nottingham NIHR BRC, Nottingham; Lifespan and Population Health, School of Medicine, University of Nottingham, Nottingham, UK; Lifespan and Population Health, School of Medicine, University of Nottingham, Nottingham, UK; Nottingham Digestive Diseases Centre, Translational Medical Sciences, School of Medicine, University of Nottingham, Nottingham, UK; Primary Care Centre Versus Arthritis, School of Medicine, Keele University, Keele, UK; Lifespan and Population Health, School of Medicine, University of Nottingham, Nottingham, UK; Academic Rheumatology, School of Medicine, University of Nottingham, Nottingham, UK; Nottingham NIHR BRC, Nottingham

**Keywords:** Pneumococcal vaccination, rheumatoid arthritis, psoriatic arthritis, vaccine safety, vaccine uptake

## Abstract

**Objective:**

The uptake and safety of pneumococcal vaccination in people with immune-mediated inflammatory diseases (IMIDs) is poorly understood. We investigated the UK-wide pneumococcal vaccine uptake in adults with IMIDs and explored the association between vaccination and IMID flare.

**Methods:**

Adults with IMIDs diagnosed on or before 1 September 2018, prescribed steroid-sparing drugs within the last 12 months and contributing data to the Clinical Practice Research Datalink Gold, were included. Vaccine uptake was assessed using a cross-sectional study design. Self-controlled case series analysis investigated the association between pneumococcal vaccination and IMID flare. The self-controlled case series observation period was up to 6 months before and after pneumococcal vaccination. This was partitioned into a 14-day pre-vaccination induction, 90 days post-vaccination exposed and the remaining unexposed periods.

**Results:**

We included 32 277 patients, 14 151 with RA, 13 631 with IBD, 3804 with axial SpA and 691 with SLE. Overall, 57% were vaccinated against pneumococcus. Vaccine uptake was lower in those younger than 45 years old (32%), with IBD (42%) and without additional indication(s) for vaccination (46%). In the vaccine safety study, data for 1067, 935 and 451 vaccinated patients with primary-care consultations for joint pain, autoimmune rheumatic disease flare and IBD flare, respectively, were included. Vaccination against pneumococcal pneumonia was not associated with primary-care consultations for joint pain, autoimmune rheumatic disease flare and IBD flare in the exposed period, with incidence rate ratios (95% CI) 0.95 (0.83–1.09), 1.05 (0.92–1.19) and 0.83 (0.65–1.06), respectively.

**Conclusion:**

Uptake of pneumococcal vaccination in UK patients with IMIDs was suboptimal. Vaccination against pneumococcal disease was not associated with IMID flare.

Rheumatology key messages

- The uptake of pneumococcal vaccination in people with immune-mediated inflammatory diseases is suboptimal.
- Vaccination against pneumococcal disease is safe.
- Pneumococcal vaccination should be actively promoted in people with inflammatory conditions.

## Introduction

Immunosuppressed adults with immune-mediated inflammatory diseases (IMIDs) such as RA, IBD and SLE are at an increased risk of pneumonia and its complications, including death [1–4]. Consequently, pneumococcal vaccination is recommended for this at-risk population [5, 6]. Despite long-standing recommendations for vaccinating the high-risk groups since the year 1992 and the over 65s since the year 2003 [5], the uptake of pneumococcal vaccination in the at-risk populations has been suboptimal [7]. In a previous study from the UK, the uptake of pneumococcal vaccination among patients with RA was reported to be 50% overall, and 43% in those younger than 65 years of age [8]. The uptake of pneumococcal vaccination across a broad range of IMIDs in a UK-wide cohort has not been evaluated to the best of our knowledge. Understanding vaccine uptake across a range of conditions is important, as the uptake of pneumococcal vaccination in people with IBD was noted to be lower in North America and Europe at 10.3–38% [9, 10].

Belief that the vaccination could trigger an IMID flare, and cause other IMIDs, e.g. vasculitis [11, 12], are key barriers to vaccination [9, 13, 14]. The association between pneumococcal vaccination and IMID flare has not been evaluated in an adequately controlled study. There is some evidence from small studies restricted to a few conditions that vaccination against pneumococcal disease does not cause a flare of IMIDs [15, 16]. In a systematic review and meta-analysis of pneumococcal vaccine immunogenicity studies in patients with SLE, disease activity did not worsen up to 8 weeks after pneumococcal vaccination [17].

In this study we evaluated the uptake and safety of pneumococcal vaccination in UK adults with IMIDs.

## Methods

### Data source

Data from the Clinical Practice Research Datalink (CPRD) Gold were used in this study. Incepted in the year 1987, CPRD Gold is an anonymized longitudinal database of electronic health records of >14 million people in the UK. CPRD participants are representative of the UK population in terms of age, sex and ethnicity [18]. CPRD includes information on demographics, lifestyle factors, diagnoses stored as Read codes—a coded thesaurus of clinical terms, primary-care prescriptions and immunizations. Vaccination and date of vaccination are also recorded.

### Approval/patient consent

This study was approved by Clinical Practice Research Datalink’s Research Data Governance (Reference 21_000614), which has overarching research ethics committee approval for research studies using anonymous data. Practices that contributed data to the CPRD consented to using anonymized patient data for approved research projects and additional consent was not required prior to individual studies (cprd.com).

### Study design

Cross-sectional and self-controlled case series (SCCS) study designs were used to examine the uptake and safety of pneumococcal vaccination respectively.

### Population

Adults aged ≥18 years on 1 September 2018, with at least one primary-care record of an IMID [i.e. RA, IBD, axial SpA (Ax-SpA), SLE] and with at least one prescription of a steroid-sparing drug (i.e. either MTX, AZA, 6-mercaptopurine, SSZ, 5-aminosalicylates, mycophenolate, LEF, ciclosporin, tacrolimus or sirolimus) within the previous 12 months were eligible to be included in this study.

### Pneumococcal vaccination

Pneumococcal vaccination was defined using both product codes and Read codes (Supplementary Table S1, available at *Rheumatology* online). Dates of vaccination were extracted from CPRD. Data on vaccination from inception of CPRD in 1987 to 1 September 2018 were considered in the vaccine uptake study. Vaccinations recorded in the CPRD as not administered in primary care, e.g. vaccination from hospitals and pharmacies were included in the vaccine uptake study but were excluded from the safety study because the date of administration is not reliably recorded in the CPRD. Similarly, in the vaccine safety study we only considered the first vaccination in those with two or more records of vaccination, as people who experience an adverse event with a vaccination are less likely to agree to have a second dose of the vaccine is offered for any clinical indication.

### Outcomes

#### Vaccine uptake

Vaccination against pneumococcal pneumonia, defined as any pneumococcal vaccination up to 1 September 2018.

#### Vaccine safety

##### Autoimmune rheumatic disease

- Primary care consultation for joint pain. This was defined using Read codes (Supplementary Table S2, available at *Rheumatology* online). Consultations for joint pain within 14 days of each other were considered as part of the same episode.
- Autoimmune rheumatic disease (AIRD) flare. This was defined as present when there was a primary-care prescription of oral CS without another CS prescription in the preceding 60 days. The patient was also required to not have consulted for an alternate condition that could justify CS prescription on the same date. For this, all relevant primary-care consultations were retrieved and reviewed by A.A. (General Medicine and Rheumatology expertise) for conditions that might explain the CS prescribed and such participants were excluded from the analysis as there was considerable uncertainty whether they experienced IMID flare or another illness (Supplementary Table S3, available at *Rheumatology* online). The CS prescription–free period used to define consultation for AIRD flare was increased to at least 120 days in a sensitivity analysis as this time period has been validated for the IBD flare [19] (see below).
- RA flare. This was defined as present when there was either a Read code for RA flare or a primary-care prescription of oral CS without another CS prescription in the preceding 60 days in patients diagnosed with RA. The patient was also required to not have consulted for an alternate condition that could justify CS prescription on the same date. This condition was applied following the same procedure as for AIRD flare described above.

##### IBD

- IBD flare. This was defined as present when there was primary-care prescription of CS without another CS prescription in the preceding 120 days [19]. The patient was also required to not have consulted for an alternate condition that could justify CS prescription on the same date defined as a new primary-care prescription of CS [19].

### Covariates

#### Vaccine uptake

Age, sex, type of IMID and presence of additional indication(s) for vaccination as per the UK Health Security Agency were considered as covariates [5]. Briefly, the additional indications for vaccination included chronic heart diseases, chronic respiratory diseases, chronic kidney diseases, chronic liver diseases, immunosuppression (defined as either solid organ transplant, bone marrow transplant, HIV/AIDS, lymphoma, leukaemia, myeloma or chemotherapy), diabetes and asplenia. The vaccine uptake study was a cross-sectional study that included patients alive on 1 September 2018. Thus, covariates were ascertained at 1 September 2018.

#### Vaccine safety

Season was the covariate of interest and was defined in line with the Meteorological Office. According to the Meteorological Office, winter spans from 1 December to the 28 February of the next year, spring spans from 1 March to 31 May, summer spans from 1 June to 31 August, and autumn spans from 1 September to 30 November.

### Statistical analyses

#### Vaccine uptake

The percentage and 95% CI of the study population alive on 1 September 2018 that was vaccinated was calculated. The proportion vaccinated was stratified according to age (<45, 45–64, ≥65 years) on the 1 September 2018, sex, presence of other indications for vaccination and type of IMID (RA, IBD, SLE, Ax-SpA). Poisson regression with robust standard error was used to examine mutually adjusted associations between pneumococcal vaccination and age group, sex, IMID type and presence of additional at-risk condition for vaccination.

#### Vaccine safety

Patients vaccinated against pneumococcal pneumonia and who also consulted their general practitioner (GP) for at-least one IMID flare in the 6-month period before and/or the 6-month period after vaccination were included in an SCCS analysis. SCCS is an established study design for assessing vaccine safety. By including patients with both an exposure and an outcome, and undertaking within-person comparisons, SCCS analysis removes between-person time-fixed confounding, a key confounder in vaccine safety studies. The baseline period extended from the latest of current registration date, first IMID diagnosis date recorded in the CPRD, and 165 days preceding vaccination to 15 days pre-vaccination, and from 90 days post-vaccination to the earliest date of 6 months post-vaccination, leaving GP surgery, death or last data collection from the GP surgery. The exposed period extended from vaccination date to 90 days later, and was further categorized as 0–14 days, 15–30 days, 31–60 days and 61–90 days (Supplementary Fig. S1, available at *Rheumatology* online). The first cut-off was selected at 14 days post-vaccination as it takes 2 weeks for the serological response, and we hypothesized that this period of immune reconstitution would be most likely to associate with disease activity. The 15-day period immediately preceding vaccination was excluded from the baseline period to minimize confounding due to healthy vaccinee effect or due to active promotion of vaccination in those consulting for a disease flare [20].

A Poisson model conditioned on the number of events adjusted for the four UK seasons as categories was fitted to calculate incidence rate ratios (aIRR) and 95% CI for each exposure period compared with the baseline period. This approach was also followed to assess the association between pneumococcal vaccination and AIRD flare, defined as a ≥4-month gap between CS prescriptions in people with autoimmune rheumatic diseases during the observation period as a sensitivity analysis.

Data management and analysis were performed in Stata v17, Stata Corp LLC, TX, USA.

## Results

### Uptake

**Figure 1.**
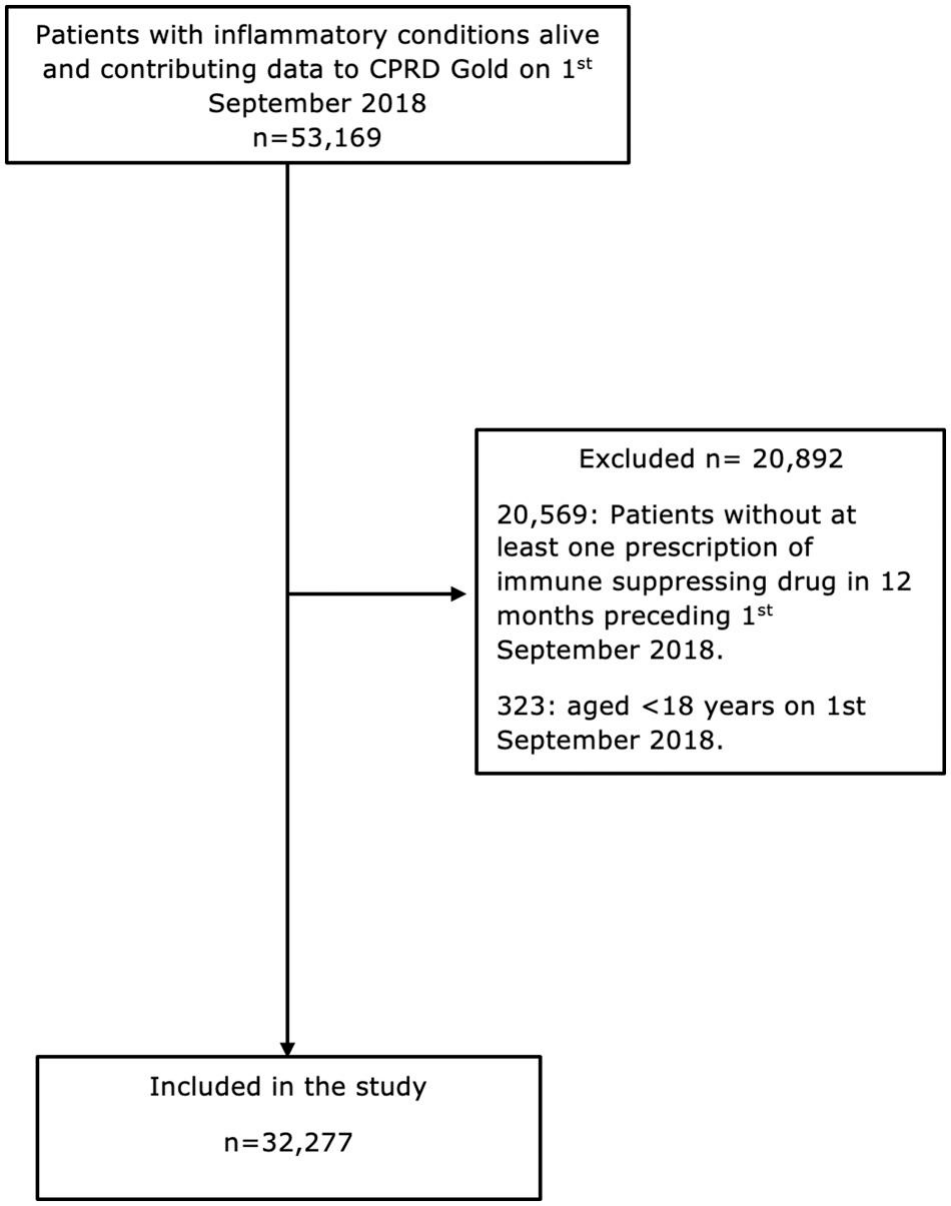
Participant selection criteria. CPRD: Clinical Practice Research Datalink

Data from 32 277 people with IMIDs were included in this study (Fig. 1). Their mean age (s.d.) on 1 September 2018 was 58 (16) years and 59% were female. A total of 14 151 (43.8%) had RA, 13 631 (42.2%) IBD, 3804 (11.8%) Ax-SpA and 691 (2.1%) SLE.

Overall uptake of pneumococcal vaccination was 56.5% (95% CI 55.9–57.0%). Pneumococcal vaccinations occurred between 1 January 1992 and 1 September 2018. Vaccination uptake was significantly lower in people with IBD [42.4%, 95% CI (41.6–43.2%)] than in those with AIRDs [66.8%, 95% CI (66.1–67.4%)], with aIRR 0.75 (95% CI 0.73–0.76). Increasing age, female sex and presence of additional at-risk conditions were independently associated with the uptake of pneumococcal vaccination (Table 1). Vaccination uptake was higher in people aged at least 65 years old or with an additional at-risk condition than in those <65 years of age and without an additional at-risk condition for vaccination [proportion vaccinated (95% CI): 0.72 (0.72–0.73) and 0.37 (0.36–0.38), respectively]. On adjusting for gender and type of inflammatory condition, people not considered at additional risk of pneumococcal pneumonia (i.e. age <65 years and without another at-risk condition) were 46% less likely to get vaccinated than those considered at additional risk of pneumococcal pneumonia (i.e. aged ≥65 years and/or with additional at-risk condition) with aIRR 0.54 (95% CI 0.53–0.56)].

**Table 1.** Uptake and risk factors of pneumococcal vaccination in immune mediated inflammatory diseases (*n* = 32 277)

		Vaccination uptake		IRR (95% CI)	
	Total number	Number vaccinated	Percent (95% CI)	Crude	Adjusted
Overall	32 277	18 227	56.47 (55.93–57.01)		
Age, years					
<45	7190	2285	31.78 (30.71–32.87)	1	1
45–64	13 045	6297	48.27 (47.41–49.13)	1.52 (1.46–1.58)	1.36 (1.31–1.42)
≥65	12 042	9645	80.09 (79.37–80.80)	2.52 (2.43–2.61)	2.07 (1.99–2.14)
Sex					
Male	13 231	7055	53.32 (52.47–54.17)	1	1
Female	19 046	11 172	58.66 (58.00–59.36)	1.10 (1.08–1.12)	1.04 (1.02–1.06)
Additional clinical risk group(s)					
Absent	19 643	9117	46.41 (45.72–47.11)	1	1
Present	12 634	9110	72.11 (71.32–72.88)	1.55 (1.53–1.58)	1.30 (1.28–1.32)
Inflammatory condition					
RA	14 151	9868	69.73 (68.97–70.49)	1	1
IBD	13 631	5779	42.40 (41.57–43.23)	0.61 (0.59–0.62)	0.75 (0.73–0.76)
SLE	691	387	56.01 (52.28–59.67)	0.80 (0.75–0.86)	0.98 (0.92–1.04)
Axial SpA^a^	3,804	2193	57.65 (56.07–59.21)	0.83 (0.80–0.85)	0.99 (0.96–1.02)

a PsA, reactive arthritis and AS. IRR: incidence rate ratio.

### Vaccine safety

Data for 1067, 935, 778 and 451 participants with primary-care consultations for joint pain, AIRD flare, RA flare and IBD flare, respectively, were included. A total of 1838 participants had either AIRD flare or a primary-care consultation for joint pain and of these, 1412 (76.8%) had RA, 281 (15.3%) had axial SpA and 145 (7.9%) had SLE. The majority were female (71.6%) and their mean (s.d.) age was 55 (12) years. Of the participants with IBD flare, 240 (53.2%) had ulcerative colitis, 160 (35.5%) had Crohn’s disease and 51 (11.3%) had IBD without any specific coding for subtype.

Vaccination against pneumococcal pneumonia was not associated with joint pain consultation, AIRD flare or IBD flares, respectively, in the 90 days post-vaccination (Tables 2 and 3). The 15-day pre-vaccination period associated with significantly more primary-care consultations for joint pain, AIRD flare and IBD flare (Tables 2 and 3).

**Table 2.** The association between pneumococcal vaccination and consultation for AIRD flare, RA flare, joint pain and IBD flare

Outcome	Risk period (days)	Events (*n*)	Person-time (days)	IRR (95% CI)	Adjusted IRR (95% CI)^a^	*P*-value
AIRD flare	Baseline	1048	373 886	1	1	
	15 days pre-vaccination	96	22 656	1.50 (1.22–1.85)	1.52 (1.23–1.88)	<0.001
	Post-vaccination intervals					
	0–90 days	391	138 045	0.99 (0.88–1.11)	1.05 (0.92–1.19)	0.514
	0–14 days	63	22 914	0.97 (0.75–1.25)	0.99 (0.77–1.28)	0.953
	15–30 days	51	22 985	0.78 (0.59–1.03)	0.80 (0.61–1.07)	0.133
	31–60 days	128	46 033	0.97 (0.81–1.17)	1.04 (0.86–1.26)	0.681
	61–90 days	149	46 113	1.13 (0.95–1.34)	1.20 (1.00–1.44)	0.050
RA flare	Baseline	856	30 5713	1	1	–/–
	15 days pre-vaccination	77	18 521	1.48 (1.17–1.87)	1.50 (1.18–1.89)	0.001
	Post-vaccination intervals					
	0–90 days	321	11 3087	0.99 (0.87–1.23)	1.04 (0.79–1.37)	0.430
	0–14 days	54	18 769	1.01 (0.77–1.33)	1.04 (0.79–1.37)	0.782
	15–30 days	42	18 835	0.78 (0.57–1.07)	0.81 (0.59–1.11)	0.184
	31–60 days	107	37 710	0.99 (0.81–1.21)	1.08 (0.87–1.33)	0.488
	61–90 days	118	37 773	1.09 (0.90–1.32)	1.17 (0.96–1.43)	0.124
Joint pain	Baseline	956	33 1904	1	1	
	15 days pre-vaccination	80	20 153	1.38 (1.09–1.73)	1.38 (1.20–1.74)	0.006
	Post-vaccination intervals					
	0–90 days	341	122 959	0.94 (0.83–1.07)	0.95 (0.83–1.09)	0.434
	0–14 days	60	20 371	1.01 (0.77–1.31)	1.02 (0.79–1.33)	0.859
	15–30 days	46	20 469	0.77 (0.57–1.03)	0.78 (0.58–1.05)	0.107
	31–60 days	99	41 020	0.82 (0.67–1.01)	0.85 (0.69–1.06)	0.143
	61–90 days	136	41 099	1.12 (0.94–1.34)	1.16 (0.96–1.40)	0.129
IBD flare	Baseline	338	125 190	1	1	–/–
	15 days pre-vaccination	35	7485	1.72 (1.22–2.44)	1.79 (1.26–2.55)	0.001
	Post-vaccination intervals					
	0–90 days	126	44 906	1.03 (0.84–1.27)	0.83 (0.65–1.06)	0.143
	0–14 days	18	7485	0.89 (0.55–1.42)	0.89 (0.55–1.43)	0.629
	15–30 days	16	7485	0.79 (0.48–1.30)	0.75 (0.45–1.25)	0.271
	31–60 days	53	14 970	1.30 (0.98–1.74)	1.07 (0.77–1.49)	0.666
	61–90 days	39	14 966	0.96 (0.69–1.34)	0.79 (0.55–1.13)	0.193

a Adjusted for season. The baseline period extended from the latest of current registration date, first disease diagnosis date recorded in the CPRD, and 6 months preceding vaccination to 15 days pre-vaccination, and from 90 days post-vaccination to the earliest date of 6 months post-vaccination, leaving GP surgery, death or last data collection from the GP surgery. IRR: incidence rate ratio; AIRD: autoimmune rheumatic disease; GP: general practitioner.

**Table 3.** The association between pneumococcal vaccination and AIRD flare^a^: sensitivity analysis

Risk period (days)	Events (*n*)	Person-time (days)	IRR (95% CI)	aIRR (95% CI)^a^	*P*-value
Baseline	225	86 797	1	1	
15 days pre-vaccination	24	5204	1.78 (1.17–2.71)	1.94 (1.26–2.97)	0.002
Post-vaccination intervals					
0–90 days	105	31 817	1.25 (0.99–1.58)	1.21 (0.92–1.58)	0.166
0–14 days	18	5204	1.30 (0.80–2.10)	1.41 (0.87–2.29)	0.167
15–30 days	13	5299	0.93 (0.53–1.63)	0.99 (0.56–1.74)	0.967
31–60 days	36	10 620	1.28 (0.90–1.82)	1.29 (0.89–1.87)	0.179
61–90 days	38	10 620	1.35 (0.96–1.91)	1.34 (0.93–1.94)	0.114

a Adjusted for season; AIRD flare defined as a ≥4 month gap between steroid prescriptions in people with AIRDs during the observation period. The baseline period extended from the latest of current registration date, first disease diagnosis date recorded in the CPRD, and 6 months preceding vaccination to 15 days pre-vaccination, and from 90 days post-vaccination to the earliest date of 6 months post-vaccination, leaving GP surgery, death or last data collection from the GP surgery. aIRR: adjusted incidence rate ratio; AIRD: autoimmune rheumatic disease; GP: general practitioner.

## Discussion

This UK-wide study has shown that approximately one in two immunosuppressed adults with IMIDs in the UK are vaccinated against pneumococcal pneumonia. This is similar to the vaccine uptake of 54–56% reported in people with chronic respiratory disease, chronic kidney disease, and diabetes requiring insulin or oral hypoglycaemic medication [21]. The vaccine uptake was even lower, at 31.8%, in those <45 years of age and at 46.4% in IMID patients without an additional indication for vaccination. The vaccine uptake ranged from 42.4% to 69.7% in IBD and RA, respectively.

This study did not find an association between pneumococcal vaccination and IMID flare requiring primary-care consultation and/or treatment. An increased risk in flare of underlying disease was observed 15 days pre-vaccination, which could be attributed to opportunistic vaccine promotion to people consulting for an IMID flare, resulting in vaccination.

It is difficult to compare our findings on vaccine uptake in IMIDs with those of previous studies, since, to our knowledge, this is the first study to assess pneumococcal vaccination uptake across many inflammatory conditions and to compare uptake between different inflammatory conditions. These low vaccination rates are concerning given the increased risk of pneumococcal disease in this at-risk population for whom vaccination is recommended, and indicates that they would benefit from targeted measures to increase pneumococcal vaccine uptake. We observed substantial variation in vaccination rates across different inflammatory diseases. This may reflect differential advice from different specialties. A 2013 UK primary-care study reported 50% pneumococcal vaccine uptake in RA patients, which was lower than the 70% pneumococcal vaccine uptake reported in the current study [8]. This improvement in pneumococcal vaccination uptake over a 5-year period is remarkable and may be related to clear guidance from the British Society of Rheumatology to offer vaccination against pneumococcal pneumonia in patients with RA [22, 23]. Similarly, more and more patients with RA are being treated with potent combination of DMARDs and this has resulted in vaccination being promoted more actively in people with this condition [24]. Improvement in pneumococcal vaccination uptake in RA patients has also been reported in a multicentre prospective study of 1679 patients in Greece [25]. In our study, patients with IBD had a low vaccine uptake. A similar low pneumococcal vaccine uptake of 38% has been reported from a French online survey of IBD patients [10] while a lower rate of 10.3% was reported from a gastroenterology clinic in Canada [9]. Pneumococcal vaccine uptake was higher in people with Ax-SpA in the current study than has been reported in other countries in Europe. This may be because Ax-SpA patients treated with NSAIDs alone were not included in this study. For comparison, in Switzerland, the pneumococcal vaccine uptake was reported to be 32.5% in an online survey of Ax-SpA patients that was not restricted to patients on immunosuppressive treatment [26]. The multinational COMOrbidities in Rheumatoid Arthritis (COMORA) study reported higher uptake of pneumococcal vaccine in some countries, e.g. France, but lower uptake in most other countries [27]. These questionnaire studies are prone to bias from self-reported vaccination uptake and should be interpreted with caution.

Consistent with previous research on factors associated with increased vaccine uptake, female sex, increasing age and other indications for vaccination significantly associated with pneumococcal vaccination [13, 28, 29].

Barriers to vaccination have included the fear that vaccines may trigger an IMID flare [14, 30]. This study did not find a significant association between pneumococcal vaccination and flare of the underlying inflammatory disease. Similarly, a systematic review and meta-analysis of pneumococcal vaccine immunogenicity studies in patients with SLE did not find an association between the vaccination and increased disease activity [17]. Safety studies in the general population have shown that pneumococcal vaccine is well tolerated [31]. Similarly, there was no association between vaccination with the seasonal influenza vaccine and AIRD flare, and no association between vaccination against COVID-19 and AIRD, IBD and psoriasis flare in previous studies [32–35]. A meta-analysis of prior uncontrolled studies reported a 2% pooled prevalence of IBD flare after vaccination, however it is not known whether the flares were temporally related or coincidental [36].

Strengths of this study included a large nationally representative sample of people with IMIDs in the UK given the almost universal registration with a GP for all UK residents [18]. We studied a wide range of IMIDs, improving the generalizability of the findings. The use of primary-care prescription and consultation data minimized recall bias on the association between vaccination and disease flares. To improve the validity of our case definition, we used a combination of diagnostic and prescription codes to ascertain people with IMIDs. Additionally, we defined IBD flares according to a validated definition [19] and we undertook a sensitivity analysis for the association between pneumococcal vaccination and AIRD flare using a validated IBD flare definition. Furthermore, to improve the outcome fidelity, we excluded participants with diagnoses that could potentially explain CS prescribed on the same date as the AIRD or IBD flare. Finally, our use of SCCS methodology controlled for between-person confounding, which is a serious problem in observational studies of vaccine safety.

There are some limitations to our study. First, some vaccinations that were administered outside of the primary care setting, for example in hospital or at the workplace as for healthcare professionals, may not have been recorded in the CPRD, reducing vaccination uptake estimates. This is unlikely to have a significant impact on our results as vaccination is almost exclusively a general practice activity in the UK. Where non-primary-care administration of the vaccine was recorded, it was excluded from the vaccine safety study as it is difficult to be sure of the date of vaccination in such instances. Second, the type of vaccine was not assessed as the vast majority of vaccinations were with the PPV23 vaccine, which has been universally used in the UK for risk groups since the year 1992 [5]. Third, we were unable to assess the impact of biologics on vaccine safety because their prescription is not recorded in the CPRD. We see no reason though to expect more extreme immunologically driven side effects in these groups given the possibility of less immunogenic response with biologic use [37]. Fourth, data on disease activity and flares managed in hospital or specialist clinics are not recorded in the CPRD. Fifth, because our definition of AIRD and IBD flare required consultation and/or prescription, minor flares not needing drug treatment were not considered as an outcome in the vaccine safety study. It is possible that there may be an association of vaccination with minor flares, which were not ascertained in our study. However, such effects would be unlikely to greatly discourage vaccination uptake and it is the more significant flares which we have studied which are of primary concern. Flares managed in hospital or specialist clinics were excluded. Additionally, joint pain was considered as an outcome of interest because it is a common symptom of inflammatory arthritis. However, joint pain might also be caused by another illness such as OA, reducing the specificity for this outcome.

In conclusion, this study provides recent UK-wide population evidence that the uptake of pneumococcal vaccination in people with IMIDs is suboptimal particularly in patients with IBD, those younger than 65 years of age, and in those without another indication for vaccination. It also demonstrated that pneumococcal vaccination does not associate with flare of the underlying IMIDs. These data should be used to promote pneumococcal vaccination in this at-risk population.

## Supplementary Material

keae160_Supplementary_Data

## Data Availability

Data used in the study are from the Clinical Practice Research Datalink (CPRD). Due to CPRD licensing rules, we are unable to share data used in this study with third parties. The data used in this study may be obtained directly from the CPRD. Study protocol is available from www.cprd.com.

## References

[1] van Aalst M , Lötsch F, Spijker R et al Incidence of invasive pneumococcal disease in immunocompromised patients: a systematic review and meta-analysis. Travel Med Infect Dis 2018;24:89–100. 29860151 10.1016/j.tmaid.2018.05.016

[2] Wotton CJ , Goldacre MJ. Risk of invasive pneumococcal disease in people admitted to hospital with selected immune-mediated diseases: record linkage cohort analyses. J Epidemiol Community Health 2012;66:1177–81. 22493476 10.1136/jech-2011-200168

[3] Shea KM , Edelsberg J, Weycker D et al Rates of pneumococcal disease in adults with chronic medical conditions. Open Forum Infect Dis 2014;1:ofu024. 25734097 10.1093/ofid/ofu024 PMC4324183

[4] Shigayeva A , Rudnick W, Green K et al; Toronto Invasive Bacterial Diseases Network. Invasive Pneumococcal Disease Among Immunocompromised Persons: implications for Vaccination Programs. Clin Infect Dis 2016;62:139–47. 26354970 10.1093/cid/civ803

[5] UK Health Security Agency. Pneumococcal: the green book, chapter 25. 2023. https://www.gov.uk/government/publications/pneumococcal-the-green-book-chapter-25 (24 November 2023, date last accessed).

[6] Lopez A , Mariette X, Bachelez H et al Vaccination recommendations for the adult immunosuppressed patient: a systematic review and comprehensive field synopsis. J Autoimmun 2017;80:10–27. 28381345 10.1016/j.jaut.2017.03.011

[7] Campling J , Vyse A, Liu H-H et al A review of evidence for pneumococcal vaccination in adults at increased risk of pneumococcal disease: risk group definitions and optimization of vaccination coverage in the United Kingdom. Expert Rev Vaccines 2023;22:785–800. 37694398 10.1080/14760584.2023.2256394

[8] Costello R , Winthrop KL, Pye SR, Brown B, Dixon WG. Influenza and pneumococcal vaccination uptake in patients with rheumatoid arthritis treated with immunosuppressive therapy in the UK: a retrospective cohort study using data from the clinical practice research datalink. PLoS One 2016;11:e0153848. 27096429 10.1371/journal.pone.0153848 PMC4838312

[9] Malhi G , Rumman A, Thanabalan R et al Vaccination in inflammatory bowel disease patients: attitudes, knowledge, and uptake. J Crohns Colitis 2015;9:439–44. 25908717 10.1093/ecco-jcc/jjv064

[10] Loubet P , Verger P, Abitbol V, Peyrin-Biroulet L, Launay O. Pneumococcal and influenza vaccine uptake in adults with inflammatory bowel disease in France: results from a web-based study. Dig Liver Dis 2018;50:563–7. 29371056 10.1016/j.dld.2017.12.027

[11] Birck R , Kaelsch I, Schnuelle P, Flores-Suárez LF, Nowack R. ANCA-associated vasculitis following influenza vaccination: causal association or mere coincidence? J Clin Rheumatol 2009;15:289–91. 19734734 10.1097/RHU.0b013e3181b55fe4

[12] Abdalla A , Sebaoui S, Alraqi S. Diffuse cutaneous reaction following PPV-23 pneumococcal vaccine: an immunisation-associated hypersensitivity vasculitis. BMJ Case Rep 2020;13:e234714. 10.1136/bcr-2020-234714 PMC7101045 32193184

[13] Loubet P , Kernéis S, Groh M et al Attitude, knowledge and factors associated with influenza and pneumococcal vaccine uptake in a large cohort of patients with secondary immune deficiency. Vaccine 2015;33:3703–8. 26073016 10.1016/j.vaccine.2015.06.012

[14] Fuller A , Hancox J, Vedhara K et al Barriers and facilitators to vaccination uptake against COVID-19, influenza, and pneumococcal pneumonia in immunosuppressed adults with immune-mediated inflammatory diseases: a qualitative interview study during the COVID-19 pandemic. PLoS One 2022;17:e0267769. 36084032 10.1371/journal.pone.0267769 PMC9462800

[15] Rezende R , Ribeiro F, Albuquerque E et al Immunogenicity of pneumococcal polysaccharide vaccine in adult systemic lupus erythematosus patients undergoing immunosuppressive treatment. Lupus 2016;25:1254–9. 26923283 10.1177/0961203316636472

[16] Migita K , Akeda Y, Akazawa M et al Opsonic and antibody responses to pneumococcal polysaccharide in rheumatoid arthritis patients receiving golimumab plus methotrexate. Medicine (Baltimore) 2015;94:e2184. 26717361 10.1097/MD.0000000000002184 PMC5291602

[17] Pugès M , Biscay P, Barnetche T et al Immunogenicity and impact on disease activity of influenza and pneumococcal vaccines in systemic lupus erythematosus: a systematic literature review and meta-analysis. Rheumatology (Oxford) 2016;55:1664–72. 27160278 10.1093/rheumatology/kew211

[18] Herrett E , Gallagher AM, Bhaskaran K et al Data Resource Profile: clinical Practice Research Datalink (CPRD). Int J Epidemiol 2015;44:827–36. 26050254 10.1093/ije/dyv098 PMC4521131

[19] Lewis JD , Aberra FN, Lichtenstein GR et al Seasonal variation in flares of inflammatory bowel disease. Gastroenterology 2004;126:665–73. 14988820 10.1053/j.gastro.2003.12.003

[20] Nakafero G , Grainge MJ, Myles PR et al Association between inactivated influenza vaccine and primary care consultations for autoimmune rheumatic disease flares: a self-controlled case series study using data from the Clinical Practice Research Datalink. Ann Rheum Dis 2019;78:1122–6. 31036623 10.1136/annrheumdis-2019-215086 PMC6691866

[21] Public Health England. Pneumococcal Polysaccharide Vaccine (PPV) coverage report, England, April 2020 to March 2021. HP Report, ed. 2022. https://assets.publishing.service.gov.uk/media/619bbc91e90e0704423dbef9/hpr1921-ppv-vc.pdf (22 November 2023, date last accessed).

[22] van Assen S , Agmon-Levin N, Elkayam O et al EULAR recommendations for vaccination in adult patients with autoimmune inflammatory rheumatic diseases. Ann Rheum Dis 2011;70:414–22. 21131643 10.1136/ard.2010.137216

[23] Singh JA , Saag KG, Bridges SL Jr et al; American College of Rheumatology. 2015 American college of rheumatology guideline for the treatment of rheumatoid arthritis. Arthritis Care Res (Hoboken) 2016;68:1–25. 26545825 10.1002/acr.22783

[24] National Institute for Health and Care Excellence (NICE). Scenario: General principles of managing adults on DMARDs. 2023. https://cks.nice.org.uk/topics/dmards/management/general-principles-of-managing-dmards/ (08 February 2024, date last accessed).

[25] Thomas K , Lazarini A, Kaltsonoudis E et al Patterns and factors associated with pneumococcal vaccination in a prospective cohort of 1,697 patients with rheumatoid arthritis. Front Med (Lausanne) 2022;9:1039464. 36698802 10.3389/fmed.2022.1039464 PMC9868611

[26] Stoffel ST , Colaninno A, Bräm R, Schwenkglenks M. Pneumococcal vaccination among adult risk patient with axial spondyloarthritis in Switzerland: data from the survey of the ankylosing spondylitis association of Switzerland (SVMB). Vaccine 2022;40:6206–10. 36175212 10.1016/j.vaccine.2022.09.056

[27] Hmamouchi I , Winthrop K, Launay O, Dougados M. Low rate of influenza and pneumococcal vaccine coverage in rheumatoid arthritis: data from the international COMORA cohort. Vaccine 2015;33:1446–52. 25659279 10.1016/j.vaccine.2015.01.065

[28] Nakafero G , Grainge MJ, Myles PR et al Predictors and temporal trend of flu vaccination in auto-immune rheumatic diseases in the UK: a nationwide prospective cohort study. Rheumatology (Oxford) 2018;57:1726–34. 29901743 10.1093/rheumatology/key156 PMC6152422

[29] Garg M , Mufti N, Palmore TN, Hasni SA. Recommendations and barriers to vaccination in systemic lupus erythematosus. Autoimmun Rev 2018;17:990–1001. 30103044 10.1016/j.autrev.2018.04.006 PMC6150817

[30] Lawson EF , Trupin L, Yelin EH, Yazdany J. Reasons for failure to receive pneumococcal and influenza vaccinations among immunosuppressed patients with systemic lupus erythematosus. Semin Arthritis Rheum 2015;44:666–71. 25701500 10.1016/j.semarthrit.2015.01.002 PMC4464934

[31] Musher DM , Manof SB, Liss C et al Safety and antibody response, including antibody persistence for 5 years, after primary vaccination or revaccination with pneumococcal polysaccharide vaccine in middle-aged and older adults. J Infect Dis 2010;201:516–24. 20092407 10.1086/649839

[32] Adams L , Nakafero G, Grainge MJ et al Is vaccination against COVID-19 associated with psoriasis or eczema flare? Self-controlled case series analysis using data from the Clinical Practice Research Datalink (Aurum). Br J Dermatol 2023;188:297–9. 36763860 10.1093/bjd/ljac061

[33] Card TR , Nakafero G, Grainge MJ et al Is vaccination against COVID-19 associated with inflammatory bowel disease flare? Self-controlled case series analysis using the UK CPRD. Am J Gastroenterol 2023;118:1388–94. 36826512 10.14309/ajg.0000000000002205

[34] Nakafero G , Grainge MJ, Card T et al Is vaccination against COVID-19 associated with autoimmune rheumatic disease flare? A self-controlled case series analysis. Rheumatology (Oxford) 2023;62:1445–50. 36048896 10.1093/rheumatology/keac484 PMC10070057

[35] Nakafero G , Grainge MJ, Myles PR et al Association between inactivated influenza vaccine and primary care consultations for autoimmune rheumatic disease flares: a self-controlled case series study using data from the Clinical Practice Research Datalink. Ann Rheum Dis 2019;78:1122–6. 31036623 10.1136/annrheumdis-2019-215086 PMC6691866

[36] Desalermos A , Pimienta M, Kalligeros M et al Safety of immunizations for the adult patient with inflammatory bowel disease-a systematic review and meta-analysis. Inflamm Bowel Dis 2022;28:1430–42. 34849941 10.1093/ibd/izab266

[37] Hua C , Barnetche T, Combe B, Morel J. Effect of methotrexate, anti–tumor necrosis factor α, and rituximab on the immune response to influenza and pneumococcal vaccines in patients with rheumatoid arthritis: a systematic review and meta‐analysis . Arthritis Care Res (Hoboken) 2014;66:1016–26. 24339395 10.1002/acr.22246

